# Protection of Renal Tubular Cells by Antioxidants: Current Knowledge and New Trends

**DOI:** 10.22074/cellj.2015.503

**Published:** 2015-01-13

**Authors:** Azar Baradaran, Hamid Nasri, Mahmoud Rafieian-Kopaei

**Affiliations:** 1 Department of Pathology, Isfahan University of Medical Sciences, Isfahan, Iran; 2 Department of Nephrology, Division of Nephropathology, Isfahan University of Medical Sciences, Isfahan, Iran; 3 Medical Plants Research Center, Shahrekord University of Medical Sciences, Shahrekord, Iran

**Keywords:** Acute Renal Injury, Kidney Injury, Antioxidants, Oxidative Stress

## Abstract

Acute renal damage mainly develops following toxic or ischemic insults and is defined as acute. These damages have largely been attributed to oxidative stress. Recently much attention has been directed toward decreased renal tubular cell regeneration during tubular cell injury. Antioxidants have recently been the focus of researchers and scientists for prevention and treatment of various oxidative stress-related conditions, including renal toxicities. Although free radicals are known to contribute in kidney injury and abundant researches, particularly laboratory trials, have shown the beneficial effects of antioxidants against these complications, long term clinical trials do not uniformly confirm this matter, especially for single antioxidant consumption such as vitamin C. The aim of this paper is to discuss the possible explanation of this matter.

Acute renal damage mainly develops following toxic or ischemic insults and is defined as acute tubular cell injury and kidney dysfunction (1-3). However, the pathogenesis of acute renal injury is complex and promoting events may be completely different, similar pathways may be concerned in subsequent injury responses (4-8). Recently much attention has been directed toward decreased renal tubular cell regeneration during tubular cell injury. Indeed, nowadays attentions are mostly on prevention, protection as well as acceleration of tubular cells regeneration against injurious insults to the kidney. To study acute kidney injury (AKI) models, various methods have been defined for each specific condition. Gentamicin (GM) derived from gram-positive bacteria has potential in treating aerobic gram-negative bacteria. However, GM has been extensively used for induction of AKI in preclinical investigations and evaluation of renal protective agents. Accumulation of GM in kidney proximal tubular cells may trigger renal injury which leads to brush border network damage (9-11). The kidney toxicity involves production and acceleration of kidneys free radical, utilization of antioxidant defense mechanisms and acute renal tubular cells necrosis (9-12), which leads to diminished glomerular filtration rate (GFR) and kidney dysfunction. The pathological mechanisms also involve up-regulation of transforming growth factor-beta (TGF-β), rise of endothelin-1, augmentation of oxidative stress, significant increase in monocyte/macrophage infiltration into the renal cortex and medulla, apoptosis and eventually necrosis (10-15). GM has also been shown to amplify the generation of superoxide anions, hydroxyl radicals, reactive oxygen species (ROS) and hydrogen peroxide in proximal tubular cells, leading to kidney damage (9, 10). Most researchers against GM renal toxicity, therefore, focused on the use of various antioxidants such as vitamins C and E or antioxidants of medicinal plants (9, 10). Indeed role of renal mitochondria against GMnephrotoxicity protection, the role of antioxidants in either protecting or mitigating GM renal toxicity, as well as integrative glomerular and tubular effects and their possible interplay have been described. Oxidative stress reflects the imbalance between the level of production and removal of cell oxidants. In oxidative stress, an increase in ROS and reactive nitrogen species (RNS) and/ or decrease in body antioxidants (exogenous/ endogenous) will happen. This imbalance suppresses the ability of biological systems in detoxification of the reactive intermediates or in repair of the resulting damage. Thus it should be noted that GM administration can easily induce severe renal toxicity, which is used to study drug-induced acute kidney damage. This complication has been attributed to generation of ROS in the kidney. In fact, AKI is a common clinical entity with high mortality and morbidity rates (8-11). A large number of patients also have other complications such as diabetes, vascular disease or chronic renal failure which put them at higher risk of AKI due to ischemic and nephrotoxic insults (11-15). Recently medicinal plants have been the focus of researchers and scientists for prevention and treatment of various oxidative stress-related conditions (8, 16). Medicinal plants possess a lot of phytochemicals with antioxidant properties including phenolic and carotenoid compounds (17, 18). Carotenoid consumption has been shown to reduce the risk of several chronic and degenerative complications (19). Phenolic compounds are abundantly presented in medicinal plants and food products and mainly consisted of anthocyanins, phenolic acids, tannins and flavonoids. These compounds possess a wide range of antioxidant activities (20, 21). Kidney damage induced by oxidative stress is associated with increased ROS/RNS production which is significantly prevented by antioxidants (8, 19-22). Medicinal plants-derived antioxidants enhance endogenous antioxidants ability to protect renal damage through reduction of lipid peroxidation (LPO) (23, 24). Tocotrienol, a member of vitamin E family with antioxidant activity, supplementation has been shown to increase glutathione (GSH) level and catalase activity and reduce renal LPO, resulting in proximal tubular injury. Moreover, it is capable of improving the index of NO_2_-/NO_3_- generation. Tocotrienol has also shown to protect the kidney damage induced by potassium dichromate (23). Ligustrazine which is an alkaloid extracted from ligusticum wallichii possesses antioxidant property. It is capable of protecting kidneys from ischemia/ reperfusion injuries by reducing malondialdehyde (MDA), decreasing ROS generation and elevating superoxide dismutase (SOD) activity. Troxerutin has been shown to reduce oxidative stress-induced kidney damage. It is abundantly found in tea, coffee, cereal grain and a variety of vegetables and fruits, while is able to reduce MDA level and enhances antioxidant enzyme activities, including SOD, glutathione peroxidase (GPx), Cu/Zn and catalase (24, 25). As mentioned, antioxidants usually act by giving electrons to free radicals and trying to turn them neutral. It has been elucidated that people who intake low vegetables and fruits are at greater risk of developing some complications. Although free radicals are known to contribute in kidney injury (26, 27), heart disease; diabetes (19-27), atherosclerosis (20-28), nephrotoxicity, hepatotoxicity, cognition (21-28), vision loss (29) and abundant researches, particularly laboratory trials, have shown the beneficial effects of antioxidants against these complications, long term clinical trials do not uniformly confirm this matter (30). It seems that the molecules which found naturally in grains, vegetables and fruits usually act to prevent a variety of conditions such as kidney injury, but not all antioxidants in different conditions act the same.

There is little evidence indicating taking single antioxidant such as vitamin C or E is clinically able to protect against kidney injury as well as other oxidative stress-related complications (28-30). The findings about consumption of antioxidant combinations are not entirely clear (30-34). In this regard, it is not clear why single or even combination of antioxidants do not act the same as antioxidants in vegetables and fruits. The possible explanation is that antioxidants usually act as parts of complicated networks, and therefore, single antioxidant cannot do the same as the whole natural products (8, 16, 30, 31, 35). Although it has been elucidated that eating grains, vegetables, and fruits which are rich in antioxidants provides protection against oxidative stress-related complications such as kidney injury, but this does not mean that antioxidants will prevent or fix the problem, especially not when they are taken out of their natural context. Notably, the results of various researches are inconclusive; however, most of the studies have had limitations such as short duration and conducting on patients with existing diseases.

## Conclusion

Oxidative stress is contributed to kidney damage by increasing oxidative stress, particularly insufficiency of endogenous antioxidant defense system. Antioxidant activity of medicinal plants has been demonstrated to prevent oxidative kidney damage by reduction of LPO and by an increase in scavenging ability of antioxidant defense system. Consumption of medicinal plants seems to be important remedies to abrogate pathology of oxidative stress-related complications, like kidney injury, while these antioxidants usually act as parts of complicated networks and therefore, single antioxidant cannot do the same as the whole natural products. Although it has been elucidated that eating grains, vegetables, and fruits provides protection against oxidative stress-related complications such as kidney injury, but this does not mean that antioxidants will prevent or fix the problem, especially not when they are taken out of their natural context.
